# Attain: Inclusive annotated pavement distress types and severity dataset

**DOI:** 10.1016/j.dib.2025.111715

**Published:** 2025-05-27

**Authors:** Mohammad Rezaeimanesh, Amir Golroo, Mohammad Sadegh Fahmani, Mohammad Javad Amani, Farid Hasanitabaar, Mohammad Saleh Entezari, Sane Karimi

**Affiliations:** Department of Civil and Environmental Engineering, Amirkabir University of Technology, Tehran, Iran

**Keywords:** Pavement defects, Deep learning, Object detection, Classification, Pavement maintenance systems

## Abstract

Pavement distress detection plays a crucial role in pavement management and rehabilitation (M&R) by providing essential data for maintenance decision-making. The Attain dataset is introduced to facilitate the development of machine learning and deep learning models for pavement distress classification and object detection. The dataset consists of 2293 images collected using three smartphones mounted on vehicle windshields. All photos are manually annotated by pavement engineer experts. The images capture a variety of pavement conditions under different lighting and traffic scenarios, making the dataset diverse and suitable for training robust models. The dataset encompasses ten distinct pavement distress categories including alligator cracks, block cracks, longitudinal and transverse (linear) cracks, faded marking, lane/shoulder drop-off, patch and utility cut, potholes, manhole, weathering, and raveling at two levels of severity i.e., low, medium, and high. In total, 19,761 distress-type instances were detected and annotated with distress type and severity. The core competency of the Attain dataset is the wide range of pavement distress types included and the severity levels of each distress provided. The use of readily available smartphone cameras significantly reduces the cost of data collection while maintaining high-quality image acquisition. The Attain dataset aims to support researchers and practitioners in developing automated pavement distress detection systems to enhance the efficiency and accuracy of pavement maintenance processes.

Specifications Table

**Table:** 

Subject	Engineering & Materials science
Specific subject area	Pavement Distress Detection and Classification
Type of data	RGB Images (.jpg), Annotation Files (.xml and .txt)
Data collection	The data were collected using three Samsung smartphones: Galaxy A72 (1920*1080), Galaxy S20 FE (3840*2160), and Galaxy S22 (3840*2160). These smartphones were mounted and fixed on the windshields of a vehicle using two phone holders. The vehicle traveled at speeds in the range of 20 to 70 km/h while the smartphone cameras were recording videos of the pavement surface. A total of 20 hours of video recordings were then used to extract images of pavement distresses. The dataset contains images with resolutions of either 640*640 or 1479*508 pixels and covers ten different pavement distress types with their severity.
Data source location	Tehran, Iran
Data accessibility	Repository name: Attain Data identification number: 10.17632/nykrzdm74f.1 Direct URL to data: https://data.mendeley.com/datasets/nykrzdm74f/1
Related research article	

## 1 Value of the Data


 

• All images in the Attain pavement distress dataset were captured using smartphones mounted on the front and rear windshields and each smartphone was stabilized by two phone holders.
• Images were taken by three different models of Samsung smartphones, Galaxy A72, S20 FE, and Galaxy S22. This variety can provide machine learning and deep learning models with images of different qualities and enhance their generalizability.
• The Attain dataset encompasses various challenges in pavement distress detection, including stains on the road surface, different lighting, and traffic conditions. It also covers different functional classifications of roads, such as local roads and highways.
• Images in the Attain dataset were captured while the vehicles’ velocity was in the range of 20 to 70 km/h, to further enhance the variety of data and improve the robustness of the models trained on this dataset.
• The Attain dataset comprises ten different pavement distress types, longitudinal and transverse cracks (linear cracks), alligator cracks, block cracks, weathering, lane/shoulder drop-off, raveling, patching and utility cuts, manholes, faded markings, and potholes.
• The cost of data collection was significantly reduced by utilizing widely accessible and conventional technologies.
• The Attain dataset is designed to support diverse machine learning tasks, such as object detection and multi-class classification. This dataset can be used to benchmark models like YOLO, Faster R-CNN, or Resnet to localize and classify pavement distress types. Additionally, we have obtained classification accuracy benchmarks from sample models (e.g., YOLO) used in our study, which achieved mAP values above 0.92 for alligator cracking detection. These results demonstrate the practical utility of our method for road agencies aiming to automate pavement condition evaluation with minimal infrastructure investment.

## 2 Background

Pavement management and rehabilitation (M&R) is a crucial and budget-intensive component of pavement management systems. Pavement distress detection can help corresponding agencies in assessing pavement quality across road networks. However, the traditional process of data collection for M&R purposes requires significant manpower, financial investment, and technological resources, and is prone to human error [1].

Therefore, automating pavement distress detection and classification can alleviate financial burdens on pavement management agencies and minimize human errors. Researchers employ machine learning and deep learning methods to detect various pavement distresses from road surface images [[2], [3], [4]]. The Attain dataset has been developed to provide high-quality images for training machine learning and deep learning models with the purpose of accurate and efficient classification and object detection of pavement distresses.

## 3 Data Description

**Fig. 1:**
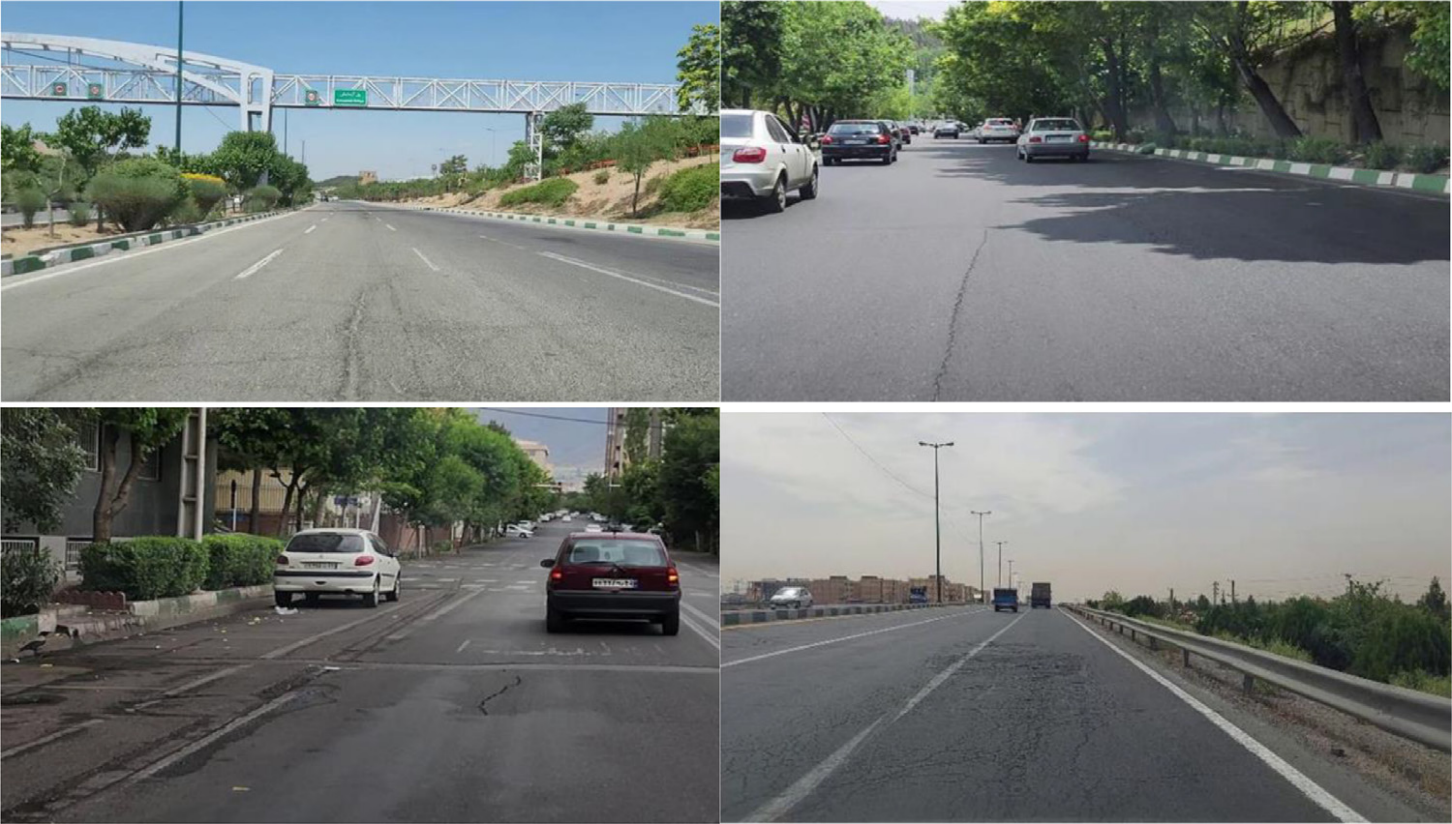
Example images of the Attain dataset.

The Attain pavement distress dataset introduced in this study includes ten categories of pavement distress: longitudinal and transverse cracks (linear cracks), alligator cracks, block cracks, weathering, lane/shoulder drop-off, manholes, faded markings, raveling, patching and utility cuts, and potholes. Example images from the dataset are shown in Fig. 1. The different lighting and traffic conditions included in the dataset are demonstrated in this figure.

**Fig. 2:**
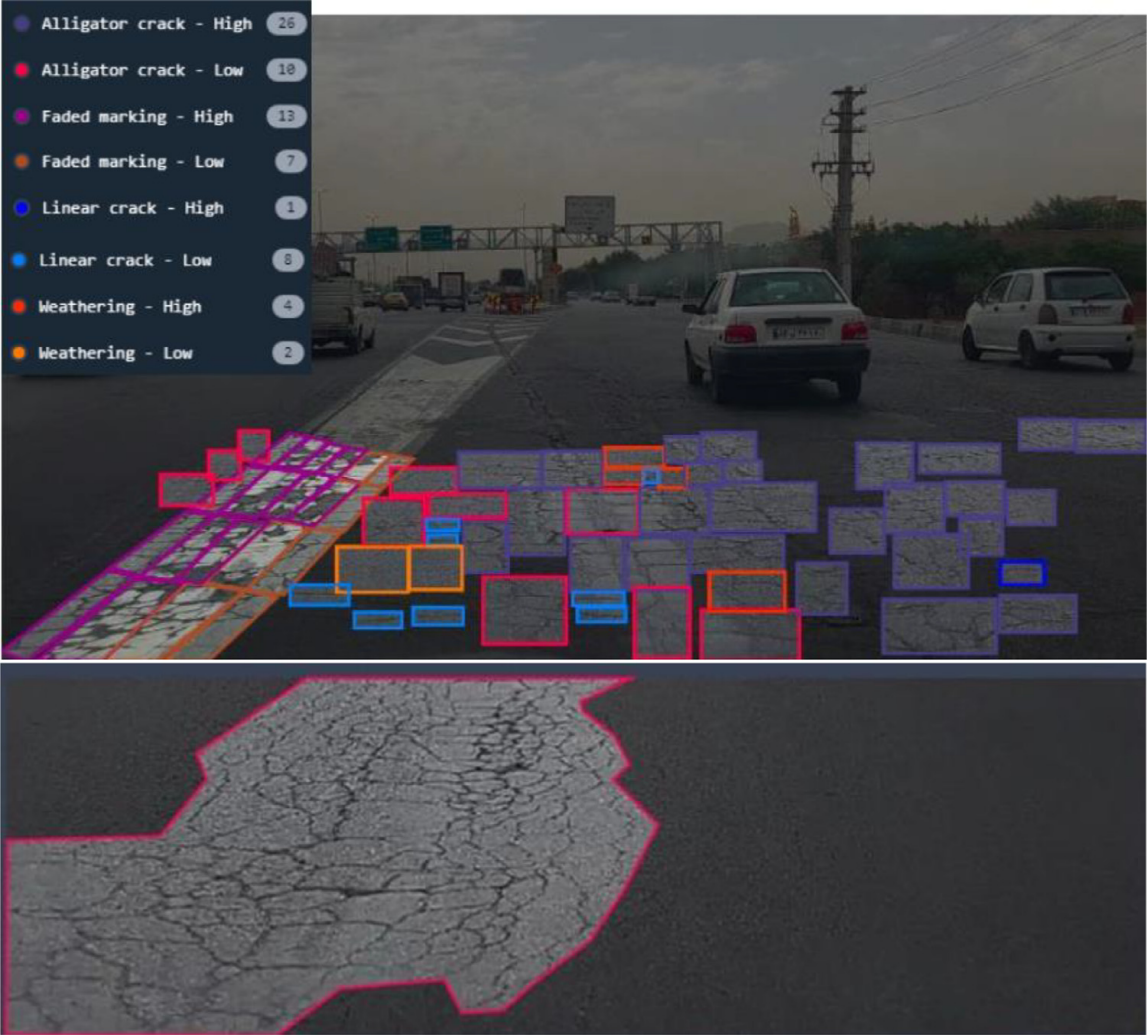
Example annotations of Attain dataset images.

The Attain dataset contains a total of 2,293 images, distributed in three separate repositories: Attain_SMP_OS_v1.0, Attain_SMP_WS_v1.0, and Attain_SMP_WS_v2.0. Details regarding the number of images and distress types in each repository are presented in Table 1, Table 2. As shown in Table 2, there are a total of 19,761 instances of pavement distress in the Attain dataset. Two examples of annotated images with the purpose of object detection and classification are illustrated in Fig. 2.

**Table 1:** Image and label characteristics of the Attain dataset.

Folder Name	No. of Images	Image Size	Annotation	Annotation Format
OS_v1.0	637	640*640	Object detection Classification	Text-based (Polygon Annotation Format)
WS_v1.0	809	640*640	Object detection Classification	Text-based (YOLO)
WS_v2.0	847	640*640 1479*508	Object detection Classification	XML-based (Pascal VOC)

**Table 2:** Number of instances of each distress in the Attain dataset.

Distress Type	Alligator Crack	Linear Crack	Block Crack	Faded Marking	Lane/ Shoulder Drop-off	Manhole	Patching	Pothole	Raveling	Weathering
OS_v1.0	198	720	-	-	-	-	-	-	-	-
WS_v1.0	1349	4103	3	94	-	504	129	210	20	721
WS_v2.0	2704	4196	58	1363	348	-	746	315	340	1640
Total	4251	9019	61	1457	348	504	875	525	360	2361

## 4 Experimental Design, Materials and Methods

**Fig. 3:**
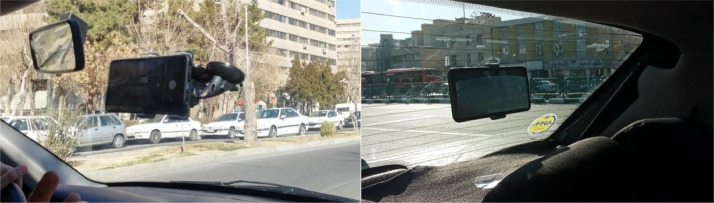
Smartphone setup on the front and rear windshields.

Images in this dataset were captured using three different Samsung smartphones. The specifications of these smartphone cameras are presented in Table 3. As shown in Fig. 3, the smartphones were mounted and fixed on the front and rear windshields inside the vehicle using two phone holders to minimize camera shaking avoiding blurred images. As the smartphones were installed, a driver started driving the car on urban roads with various functional classifications while the cameras were recording a continuous video of the road surface. The smartphones were slightly tilted downward to capture as much of the pavement surface as possible while avoiding capturing any part of the vehicle.

**Table 3:** Camera specifications.

Smartphone	Resolution	Focal Length	Sensor Size	Phase Detection Auto Focus	Optical Image Stabilization
Galaxy A72	1920*1080	26 mm	1/1.7”	Yes	Yes
Galaxy S20 FE	3840*2160	26 mm	1/1.76”	Yes	Yes
Galaxy S22	3840*2160	24 mm	1/1.56”	Yes	Yes

The data collection was conducted in Tehran, Iran, where approximately 20 hours of video footage was recorded. During data collection, the speed of the vehicle was in the range of 20–70 km/h. To extract images from these recordings, frames were captured every 250 ms. A total of 2293 images were selected for labeling based on image quality and pavement visibility. Due to the high camera resolutions, these images were then resized to 640*640 and 1479*508 pixels. The dataset includes images taken under different lighting and traffic conditions to better simulate real-world scenarios.

## Limitations

The dataset was collected in a single city (Tehran, Iran), which may limit its generalizability to other regions with different pavement conditions and climate factors. Additionally, only three Samsung smartphone models were used for data collection, which may introduce biases related to camera quality and sensor characteristics. Although various lighting and traffic conditions are included, extreme weather conditions such as heavy rain, snow, and fog were not considered. Furthermore, only ten pavement distress types were captured in this dataset with one sensor type which was a smartphone camera. The future work would be collecting all 20 asphalt distress types introduced by ASTM D 6433 [5] using a multi-modal approach via using other types of cameras i.e., active camera, stereo-vision camera, 360 camera, drone camera, and LIDAR.

## Ethics Statement

We, the authors of this work, hereby declare that we have thoroughly reviewed and complied with all ethical standards required for publication in Data in Brief. We also confirm that this research does not involve human participants, animal testing, or the use of any data obtained from social media platforms. Our study adheres to all ethical guidelines and safeguards, ensuring the responsible and ethical execution of this research.

## CRediT Author Statement

**Mohammad Rezaeimanesh**: Data Curation, Writing - Original Draft; **Amir Golroo**: Methodology, Conceptualization, Supervision, Validation, Writing - Review & Editing; **Mohammad Sadegh Fahmani**: Data Curation; **Mohammad Javad Amani**: Investigation; **Farid Hasanitabaar**: Data Curation, Investigation; **Mohammad Saleh Entezari**: Data Curation, Investigation; **Sane Karimi**: Data Curation, Investigation.

## Data Availability

Mendeley DataAttain (Original data)
